# Forward Collision Warning Strategy Based on Millimeter-Wave Radar and Visual Fusion

**DOI:** 10.3390/s23239295

**Published:** 2023-11-21

**Authors:** Chenxu Sun, Yongtao Li, Hanyan Li, Enyong Xu, Yufang Li, Wei Li

**Affiliations:** 1 School of Mechanical and Automotive Engineering, Guangxi University of Science and Technology, Liuzhou 545616, China; 221068242@stdmail.gxust.edu.cn (C.S.); liyongtao@gxust.edu.cn (Y.L.); 2 School of Automation, Guangxi University of Science and Technology, Liuzhou 545616, China; 3 Dongfeng Liuzhou Motor Company, Liuzhou 545616, China; xuey@dflzm.com (E.X.); liyufang@dflzm.com (Y.L.); liwei1@dflzm.com (W.L.)

**Keywords:** collision warning, adaptive extended Kalman filter, millimeter wave radar, sensorfusion, YOLOv5 algorithm, attention mechanism

## Abstract

Forward collision warning (FCW) is a critical technology to improve road safety and reduce traffic accidents. However, the existing multi-sensor fusion methods for FCW suffer from a high false alarm rate and missed alarm rate in complex weather and road environments. For these issues, this paper proposes a decision-level fusion collision warning strategy. The vision algorithm and radar tracking algorithm are improved in order to reduce the false alarm rate and omission rate of forward collision warning. Firstly, this paper proposes an information entropy-based memory index for an adaptive Kalman filter for radar target tracking that can adaptively adjust the noise model in a variety of complex environments. Then, for visual detection, the YOLOv5s model is enhanced in conjunction with the SKBAM (Selective Kernel and Bottleneck Attention Mechanism) designed in this paper to improve the accuracy of vehicle target detection. Finally, a decision-level fusion warning fusion strategy for millimeter-wave radar and vision fusion is proposed. The strategy effectively fuses the detection results of radar and vision and employs a minimum safe distance model to determine the potential danger ahead. Experiments are conducted under various weather and road conditions, and the experimental results show that the proposed algorithm reduces the false alarm rate by 11.619% and the missed alarm rate by 15.672% compared with the traditional algorithm.

## 1. Introduction

With the growth of the automotive industry and the increase in the number of vehicles, there has been a rise in both the frequency and severity of traffic accidents [1]. This presents a significant danger to both personal safety and property. Vehicle collisions and rear-end collisions are among the most common traffic accidents [2,3]. Advanced assisted driving systems (ADAS) are crucial in mitigating traffic accidents and minimizing casualties. FCW is one of the ADAS technologies that continuously monitors surrounding road conditions through sensors [4]. It effectively informs the driver in a timely manner to prevent collisions with other vehicles or obstacles. In terms of safety concerns, collision warning is a crucial function of advanced driver assistance systems. The primary objective of collision warning is obstacle detection. Consequently, gaining precise object detection in complex situations is one of the main challenges for high-level autonomous automobiles.

In order to achieve target detection accuracy, many research groups and companies have intensively studied various sensor systems such as radars, cameras, light detection and ranging (LiDAR) sensors, ultrasonic radar sensors, and infrared sensors [5,6,7]. Due to the inherent limitations of the sensor, autonomous vehicles cannot meet the demands of environmental perception by relying on a single sensor in different weather conditions and complex road scenarios [8]. Collision warning systems based on radar or cameras fall short of meeting the safety requirements for road driving due to the risk of false alarms and inaccurate judgment.

Although LIDAR can provide high-resolution 3D point clouds, it is susceptible to atmospheric scattering and absorption. In addition to this, it requires sophisticated techniques such as mechanical scanning or optical coherence, which results in its relatively high cost [9,10]. Camera sensor-based target detection is a state-of-the-art technique that leverages the power of cameras to acquire high-resolution images or videos of the dynamic environment and then employs sophisticated computer vision algorithms for robust and accurate identification and localization of targets of interest [11]. Deep learning [12] has effectively leveraged its capabilities in object detection algorithms in the area of computer vision. Currently, based on the existence of the Region Proposal [13] to be chosen, deep learning may be broadly divided into two groups: one-stage object detection algorithms like R-CNN [14], SPP-net [15], Fast R-CNN [16], etc., and two-stage object detection algorithms like YOLO [17], SSD [18], etc. Zhang et al. [19] proposed an improved YOLOv5 vehicle detection algorithm, which can improve the accuracy of vehicle detection and reduce the false detection rate in different traffic scenarios. Zheng et al. [20] optimized the structure of the Fast R-CNN convolution to improve the accuracy of object recognition. Lin et al. [21] fused the attention module CBAM with the backbone part of the YOLOv5s network and proposed a fruit detection method based on the improved YOLOv5s algorithm.

In the practical application of low-end commercial vehicles and the needs of small automobile enterprises, LiDAR is difficult to mass-produce in low-end commercial vehicles due to its expensive cost and complex technology. However, millimeter-wave radar can replace LiDAR applications to a certain extent. On the one hand, millimeter-wave radar is more penetrating than LIDAR and can work in rain, fog, snow, and other bad weather. On the other hand, millimeter-wave radar costs less than LIDAR, which is ideal for low-end vehicles. Similar to LIDAR, the camera is also significantly influenced by environmental factors. Nevertheless, this limitation can be overcome by employing millimeter-wave radar. However, it also has several limitations, such as a low capacity to classify objects and sensitivity to electromagnetic interference [22]. Therefore, millimeter-wave radar and camera complement each other effectively through fusion, and the cost of the two is more affordable than that of LIDAR. Kalman filters are widely used for target tracking in millimeter-wave radar, as they can estimate the state of a target based on noisy and incomplete measurements. One of the challenges in radar target tracking is handling uncertainties and faults in the system and measurement models, such as varying noise covariances and measurement outliers. To cope with this problem, several adaptive and robust methods have been developed to tune the noise covariance or reject the outliers based on some criteria. Montañez et al. [23] applied an extended Kalman filter (EKF) to detect moving targets in a constant rotational speed and rate velocity (CTRV) kinematic model. Pearson et al. [24] presented an angle-channel Kalman filter that incorporates measures of range, range rate, and on-board dynamics to estimate the target state.

Millimeter-wave radar and visual decision-level fusion is a method for object detection in autonomous driving that combines the outputs of millimeter-wave radar and vision sensors to enhance the precision of the detection system. Liu et al. [25] proposed a novel multi-sensor decision-level fusion algorithm that combines the advantages of millimeter-wave radar and cameras for object detection and recognition in autonomous driving scenarios. Jiang et al. [26] proposed a decision-level fusion algorithm for radar detection results and image detection results and used the proposed adaptive KCF radar tracking algorithm to track the target. Intersection of Union (IoU) is a metric that measures the similarity between two regions, such as radar points and image pixels, that correspond to the same object. I It is widely used in millimeter-wave radar and vision fusion. Zhou et al. [27] reviewed the existing methods of radar and camera fusion based on IoU and classified them into three categories: data-level, decision-level, and feature-level fusion methods. Lin et al. [28] used a deep learning-derived object association method to estimate the IoU between the radar and image regions and a multi-object tracking algorithm to track the detected objects. The safety distance model is a mathematical formula that calculates the minimum distance that a vehicle should maintain from the preceding vehicle or object based on factors such as relative speed, road condition, driver reaction time, and braking performance. Different safety distance models have been proposed by researchers to account for various scenarios. Dong et al. [29] developed a coupling safety distance model for vehicle active collision avoidance, which considered the characteristics of the driver and the vehicle dynamics. Alsuwian et al. [30] used optimal control theory and acceleration compensation to calculate the longitudinal minimum safe distance. Liu et al. [31] proposed a distance and acceleration-compensated safe distance model that used predictive control as well as speed and distance prediction to determine the optimal safe distance.

Current research on forward collision warning based on millimeter-wave radar and vision fusion faces several challenges. For vision, the difficulties include dealing with road situations, the dynamics of the scenes, and changing lights. In addition, the algorithm design should ensure high robustness and real-time performance [32]. However, some existing methods may have low accuracy, high computational cost, or poor generalization ability [33]. For radar target tracking, the estimation algorithm may be challenged by modeling errors, clutter interference, and maneuvering targets. As a result, biased or inaccurate results may be produced [34]. Furthermore, some existing methods may struggle with multiple targets, nonlinear motion, or data association problems. For sensor fusion, the existing algorithms have limitations in exploiting the complementary information from radar and vision [35]. On one hand, they have low robustness and low tolerance for sensor noise, failure, errors, etc. On the other hand, they are poorly generalized and weakly adaptable to different scenarios and environments.

**Figure 1:**
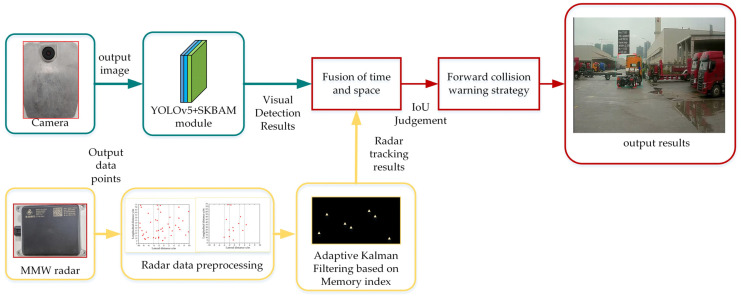
Flowchart of the forward collision warning strategy in this paper.

For these issues, this study provides a collision warning method based on millimeter-wave radar and vision fusion in complex situations. This strategy consists of four parts: radar detection, vision detection, radar and vision fusion, and collision warning strategy, respectively, as shown in Figure 1. The main contributions of the present work are as follows:

- According to the advantages of the existing advanced attention mechanism, this paper improves the CBAM attention mechanism (convolutional block attention module) and obtains a selective kernel and bottleneck attention mechanism (SKBAM). We add the SKBAM module to the network structure of the YOLOv5 algorithm model and verify the advantages of the improved YOLOv5 algorithm through an ablation experiment comparison.
- A memory index adaptive Kalman filter algorithm based on information entropy is proposed, which can adaptively adjust the noise covariance according to the system state and improve the accuracy of target tracking in millimeter-wave radar.
- A forward collision warning strategy for decision-level fusion of millimeter-wave radar and vision is designed. Experiments show that the strategy reduces the false alarm rate and missed alarm rate of the existing fusion algorithm in different environments and improves the accuracy of collision warning.

## 2. Visual Sensor Detection Model

### 2.1. YOLOv5 Visual Detection Model

**Figure 2:**
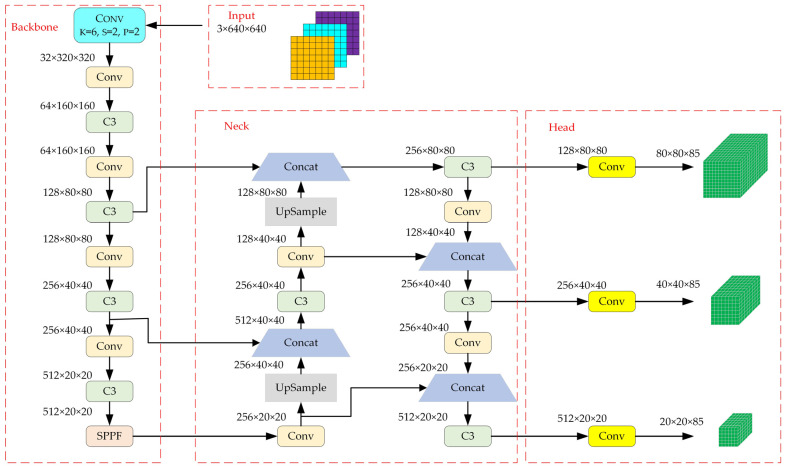
YOLOv5s version 6.0 structure.

YOLOv5 is a fast and accurate object detection model in computer vision that aims to locate and identify objects of interest in images and videos. It utilizes the concept of a grid to divide the feature map into multiple lattices. These cells predict the objects in their own regions to obtain the bounding boxes, confidence scores, and class probability maps needed to achieve object recognition. According to different complexity and performance, YOLOv5 consists of four model sizes: YOLOv5s, YOLOv5m, YOLOv5l, and YOLOv5x [36]. Among these models, YOLOv5s has the lightest and most concise network, and its inference speed is the fastest [37]. Therefore, YOLOv5s is used as the object detection model based on deep learning in this paper. There are three components to the network structure of YOLOv5s: the backbone, neck, and head [38]. Among them, the backbone of the YOLOv5-6.0 version consists primarily of the Conv module, C3 module, and SPPF module, and its model structure is shown in Figure 2.

### 2.2. Improved YOLOv5 Visual Detection Model

#### 2.2.1. Attention Mechanism

YOLOv5 is a state-of-the-art object detection model that can achieve high accuracy and speed on various datasets. However, it still faces some challenges, such as detecting small objects, handling complex backgrounds, and capturing global information. To address these issues, some researchers have proposed using attention mechanisms to enhance YOLOv5’s performance. Attention mechanisms are powerful techniques that enable a neural network to learn how to assign different weights to different inputs or outputs based on their importance or relevance. They can enhance the representation ability of the network and improve its performance on various tasks. There are different types of attention mechanisms, such as self-attention, squeeze-and-excitation networks (SENets) [39], spatial attention [40], and coordinate attention [41].

CBAM (Convolutional Block Attention Module) is an attention mechanism that can improve the performance of object detection models by refining the feature maps with channel and spatial attention modules [42]. The principle of CBAM is shown in Figure 2. CBAM consists of two modules: channel attention and spatial attention. Channel attention concentrates on the inter-dependencies among channels, and spatial attention concentrates on the inter-dependencies among spatial locations. CBAM can help the model to learn more discriminative features and enhance its detection accuracy.

**Figure 3:**
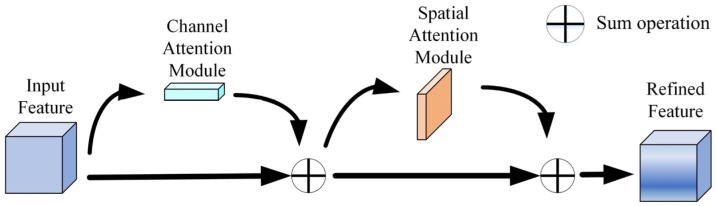
The principle of CBAM.

According to Figure 3, the input feature map is initially fed into the channel attention sub-module for computation, and then the computed attention weight is multiplied with the input feature map at the pixel level to achieve the weighted outcome. After that, it enters the spatial attention sub-module for the same operation mentioned above and finally obtains the adjusted feature map.

The operating principle of the channel attention submodule is that the feature map *F* is input into the channel attention module, which extracts the information on the channel dimension in two ways: global average pooling and global max pooling. Then a shared multi-layer perceptron (MLP) is used to generate the channel attention weight *M_C_*. Finally, *F* and *M_C_* are multiplied to obtain the channel attention-adjusted feature map *F*’, whose dimensions are consistent with the input feature map.

#### 2.2.2. Improved Channel Attention

Due to the structural defects of the traditional CBAM module, it will increase the amount of calculation and reduce the effect of the model. Inspired by the SKNet module [43] and the BAM module [44], this paper introduces the Selective Kernel and Bottleneck Attention Module (SKBAM). The channel attention mechanism submodule of CBAM is similar to SENet, which uses the attention mechanism to strengthen the feature representation of an image. SKNet enables each neuron to adjust its receptive field size dynamically according to the input information from multiple scales. SKNet makes an improvement on SENet, which can adaptively select the more important convolution kernel size than others. It can merge feature information from various scales to achieve improved performance in image segmentation, super-resolution, and other tasks. Therefore, the SKNet module replaces the channel attention mechanism in this paper. In order to make the model have better accuracy and efficiency than the original one, we propose an improved SKNet attention mechanism.

**Figure 4:**
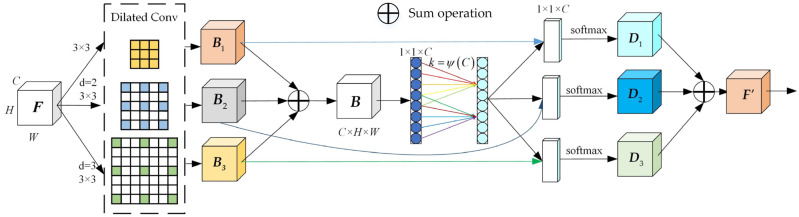
Improved SKNet structure.

The proposed improved SKNet block consists of three aspects: Split, Fuse, and Select, which are the same as the traditional module. The improved SKNet structure is shown in Figure 4.

**Figure 5:**
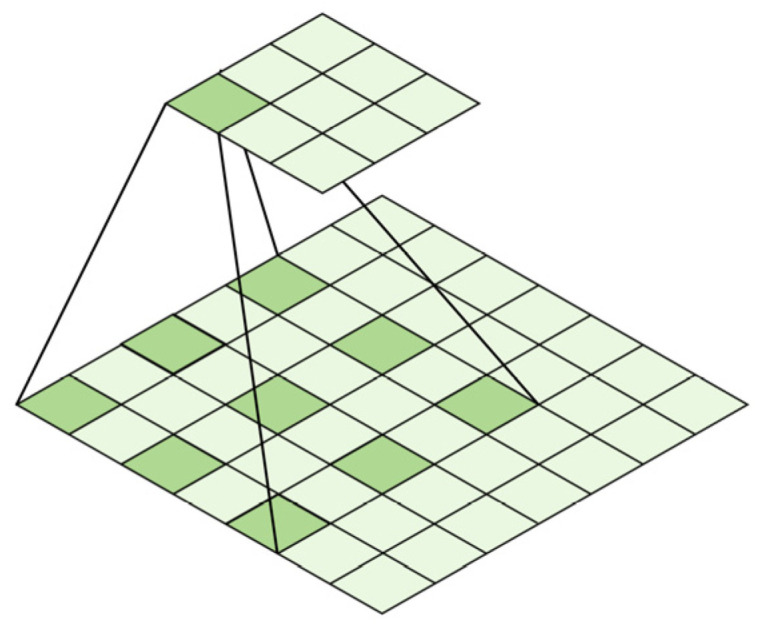
Dilation = 2, 3 × 3 dilated convolution.

The split operation convolves the input feature map using multiple convolution kernels to form three branches with kernels of size 3 × 3, 5 × 5, and 7 × 7. However, general convolution has a small receptive field, which means it can capture a little contextual information from the input. In order to enlarge the receptive field of the convolutional neural network without increasing the number of parameters, dilated convolution is used instead of conventional convolution. Figure 5 shows an example of a dilated convolution with a 3 × 3 kernel and a dilation rate of 2. The receptive field of this dilated convolution is equivalent to that of a conventional convolution with a 5 × 5 kernel, but with fewer parameters and computations. Similarly, a dilated convolution with a 3 × 3 kernel and a dilation rate of 3 has the same receptive field as a conventional convolution with a 7 × 7 kernel. Therefore, these two ordinary convolutions can be replaced by dilated convolutions.

To combine and aggregate the information from different branches with different kernel sizes, the fuse operation is used. The fused feature map ***B*** is obtained by adding the feature maps in the three branches element-wise, as shown in (1).

(1) $$B=B_{1}+B_{2}+B_{3}$$

The channel statistics ***S*** are generated by applying global average pooling to ***B*** to embed the global information. Next, ***S*** is subjected to fully connected layer operations to generate the compressed feature map ***Z***. However, fuse operation uses a fully connected layer for dimensionality reduction, which leads to the loss of some information and introduces a large number of parameters and calculations. Refer to the ECA module for the idea of improving the CENet block. We use a 1D convolution operation with a convolution kernel size of *k* to replace the fully connected layer in the fuse operation:

(2) $$W={C1D}_{k}(B)$$

where W is the parameter matrix of *C* × *C*, C1D represents the one-dimensional convolution, and *k* is the size of the convolution kernel, which can be obtained by adaptively:

(3) $$k=\psi (C)={\left|\frac{\log_{2}(C)}{\gamma }+\frac{b}{\gamma }\right|}_{odd}$$

where *C* is the channel dimension, ${\left|t\right|}_{odd}$ denotes the closest odd number to *t*, and *γ* is set to 1, *b* is set equal to 2.

The select operation performs a weighted sum of the branches using the attention vector, which yields a fused feature map with adaptive receptive field sizes for each neuron. It performs a Softmax operation at each position on the channel weight vector of the last two branches of Fuse:

(4) $$m=\frac{e^{MW}}{e^{MW}+e^{NW}+e^{CW}},\;n=\frac{e^{NW}}{e^{MW}+e^{NW}+e^{CW}},\;j=\frac{e^{JW}}{e^{MW}+e^{NW}+e^{JW}},\;m+n+j=1$$

where *m*, *n*, and *j* are the soft attention vectors of $B_{1}$**,** $B_{2}$**,** $B_{3}$, respectively. We perform element-wise operations on the split convolved features with the three vectors:

(5) $$D_{1}=m\times B_{1},\;D_{2}=n\times B_{2},\;D_{3}=j\times B_{3}$$

Finally, the output ***D*** is obtained by summing the three branches:

(6) $$F^{\prime }=D_{1}+D_{2}+D_{3}$$

#### 2.2.3. Improved Spatial Attention Submodule of CBAM

**Figure 6:**
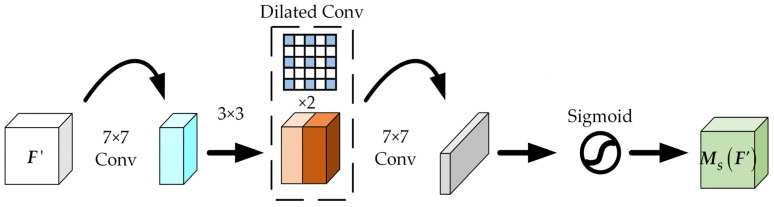
The structure of the improved spatial attention mechanism.

Pooling operations in the spatial attention submodule lead to the loss of some local detail changes in the input feature map. To overcome this deficiency, we take a page from the Bottleneck Attention Module (BAM) and remove the pooling operation. The structure of the improved spatial attention mechanism is shown in Figure 6.

Specifically, the feature map ***F***′ of size *C × W × H* obtained from the spatial attention submodule is used as the input feature map of this module. The spatial attention of the original BAM uses a 1 × 1 convolution structure. We use 7 × 7 convolutions instead of 1 × 1 convolutions because we want to capture the inter-spatial relationship of features in a larger receptive field. The input feature map $F^{\prime }\in R^{C\times H\times W}$ is first fed into a 7 × 7 convolution layer to reduce the channel dimension and obtain a compressed feature map $F_{C}^{\prime }\in R^{C/r\times H\times W}$, *r* is the reduction ratio for which the channel branches are identical. Two dilated convolutional layers with a kernel size of 3 × 3 are applied on $F_{C}^{\prime }$ to enlarge the receptive field of the attention map. Then, after another convolution operation with a convolution kernel of 7, the number of reduction channels is consistent with the input. Finally, the spatial attention map $M_{S}\left(F^{\prime }\right)\in R^{C\times H\times W}$ is generated by normalizing with the sigmoid function. This spatial attention module is summarized as follows:

(7) $$M_{s}(F^{\prime })=\sigma \left(f_{3}^{7\times 7}\left(f_{2}^{3\times 3}\left(f_{1}^{3\times 3}\left(f_{0}^{7\times 7}(F^{\prime })\right)\right)\right)\right)$$

#### 2.2.4. YOLOv5 Introduces SKBAM

**Figure 7:**
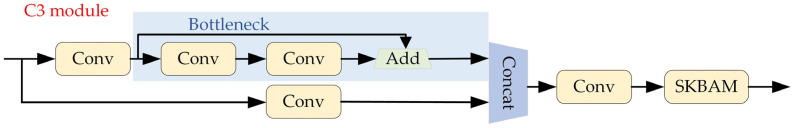
The SKBAM module is in the specific location of the C3 module.

To enhance the performance of yolov5s, the devised SKBAM is integrated into the architecture of yolov5s. The C3 module consists of three main components: a bottleneck layer, a cross-stage partial (CSP) connection, and a shuffle attention layer. The bottleneck layer is a residual block that reduces the number of channels in the input feature map by a factor of two, applies a 3 × 3 convolution, and then restores the number of channels. The CSP connection splits the output of the bottleneck layer into two branches: one branch passes through a series of bottleneck layers and then concatenates with the other branch, while the other branch passes through a 1 × 1 convolution and acts as a skip connection. The shuffle attention layer is a self-attention mechanism that reorders the channels of the feature map based on their importance and correlation. Our proposed SKBAM module is inserted into the backbone part C3 module, as detailed in Figure 7. The SKBAM module can realize the adaptive adjustment of the feature map and improve the accuracy of object detection.

## 3. Millimeter Wave Radar Detection Model

### 3.1. Radar Data Preprocessing

Millimeter-wave radar is a type of sensor that can achieve high-precision target detection and recognition in intricate environments. However, radar output often contains many interference objects, such as empty, false, or non-hazardous targets. These objects impair the performance and accuracy of subsequent processing and increase computation and resource consumption. Therefore, it is very necessary to preprocess the output data of millimeter-wave radar and remove the interference target. The specific steps are as follows:

Empty target removal: Empty targets denote target points with zero range and relative velocity, which have no practical value and are merely caused by noise or error in the radar system. Therefore, null target removal can be realized by traversing all target points and deleting them if their ranges and relative velocities are 0.

False target removal: False targets refer to target points that are beyond the radar detection range or do not conform to the physical law, which may be caused by interference signals or other reasons. Therefore, if the relative velocity of the target points is greater than the maximum radar detection speed or less than the minimum radar detection speed, they are removed. The detection speed of millimeter-wave radar ranges from −66 m/s to 66 m/s.

**Figure 8:**
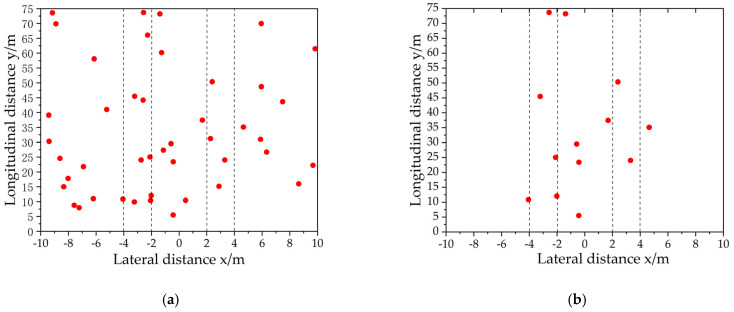
Data preprocessing process of millimeter Wave radar. The red dots in the figure are the radar output, (**a**) is the original data output from the radar, and (**b**) is the data with false and invalid noise removed by the preprocessing algorithm.

Non-hazardous target removal: Non-hazardous targets are target points that are not related to the radar system or do not pose a threat, which may be caused by background clutter or other reasons. Therefore, target points are removed if their lateral distance is greater than a set threshold or less than a negative threshold. Similarly, if their relative velocity is greater than a set threshold or less than a negative threshold, they are also removed. The thresholds of lateral distance and relative velocity of valid data points are set as follows:

(8) $$\left|x\right|\leq X_{0},\;-34\;m/s\leq v\leq 10\;m/s$$

where x is the relative lateral range of the radar detection object; $X_{0}$ is the lateral distance threshold of valid data points; and *v* is the relative velocity of the radar to detect the object. The results of radar data preprocessing are shown in Figure 8. According to the above steps, false targets are well removed.

### 3.2. Adaptive Kalman Filtering Based on Memory Index

Extended Kalman Filter (EKF) is a recursive algorithm that estimates the state of a target based on a nonlinear system model and a measurement model. The EKF linearizes the nonlinear functions around the current state estimate and applies the standard Kalman filter equations. The EKF consists of two steps: prediction and update [45].

The prediction and target state estimation steps are as follows:

(9) $${\hat{X}}_{k+1|k}=A{\hat{X}}_{k|k}+W_{k}$$

(10) $$P_{k+1|k}=AP_{k|k}A^{T}+Q_{k}$$

(11) $$Z_{k+1|k}=H{\hat{X}}_{k+1|k}+V_{k}$$

Update the target state estimate as follows:

(12) $${\hat{X}}_{k+1|k+1}={\hat{X}}_{k+1|k}+K_{k+1}[Z_{k+1}-H{\hat{X}}_{k+1|k}]$$

(13) $$K_{k+1}=P_{k+1|k}H_{k+1}^{T}{\left(H_{k+1}P_{k+1|k}H_{k+1}^{T}+R_{k+1}\right)}^{-1}$$

(14) $$P_{k+1|k+1}=(I-K_{k+1}H_{k+1})P_{k+1|k}$$

The performance of the Kalman filter depends on the precise knowledge of the process noise covariance ***Q*** and the measurement noise covariance ***R***, which are often unknown or hard to obtain in practice [46]. Ideally, they should follow the Gaussian random vector distribution. However, this assumption may not be valid in reality due to modeling errors or parameter uncertainties. If ***Q*** and ***R*** are not appropriately selected, the Kalman filter may yield biased or inconsistent estimates or even deviate from the true state. To address this problem, we propose a method of using a memory index based on information entropy for adaptive Kalman filter radar target tracking, which can dynamically adjust ***Q*** and ***R*** according to the state variation in the system.

The memory index refers to the extent to which historical observations or state vectors are memorized. The amount of information in the observation data or the state vectors reflects the uncertainty of the system state estimation. If the information amount is large, the observation data or the state vectors are less reliable, and thus more weight should be assigned to the newer ones, resulting in a smaller memory index. Conversely, if the information amount is small, the observation data or the state vectors are more reliable, and thus more weight should be assigned to the older ones, resulting in a larger memory index. Memory index based on information entropy can be expressed as:

(15) $$\alpha_{k}=\frac{1}{1+\eta_{k}S_{k}}$$

where $\eta_{k}$ is a positive adjustment parameter, which determines the sensitivity of memory index to information entropy, $\eta_{k}$ is larger, it means that it is more sensitive to information entropy, and the memory index is easier to change; $\eta_{k}$ is smaller, it means that it is less sensitive to information entropy, and the memory index is more stable. $S_{k}$ is the information entropy at time *k*, which is the information amount of observation data or state vectors, and is given by:

(16) $$S_{k}=-\sum_{i=1}^{n}p_{i}\log p_{i}$$

To compute the information entropy in the data, use the need for each dimension or weight for probability distribution and the calculation of information entropy, and then sum the total information entropy. where $p_{i}$ is the probability distribution of the *i*th observation or state vector value, which can be approximated as a Gaussian distribution and is given by:

(17) $$p_{i}=\frac{1}{\sqrt{2\pi }\sigma_{i}}\exp\left(-\frac{{\left(\beta_{i}-\mu_{i}\right)}^{2}}{2\sigma_{i}^{2}}\right)$$

where $\beta_{i}$ is the *i*th observation data or state vector of values, $\mu_{i}$ and $\sigma_{i}$ are the mean and standard deviation of the *i*th observation data or state vector value.

The innovation sequence is the result of the discrepancy between the actual measurements and the predicted measurements of the Kalman filter. It reflects the discrepancy between the model and reality, and can be used to estimate the actual measurement noise. The innovation sequence is as follows:

(18) $${\tilde{y}}_{k}=Z_{k}-H_{k}{\hat{X}}_{k|k-1}$$

The covariance matrix of the innovation sequence $C_{k}$ is as follows:

(19) $$C_{k}=H_{k}P_{k|k-1}H_{k}^{T}+R_{k}$$

The residual sequence $e_{k}$ is defined as the difference between the actual measurement and the updated estimate of the Kalman filter. The residual sequence $e_{k}$ is as follows:

(20) $$e_{k}=Z_{k}-H_{k}{\hat{X}}_{k|k}$$

In (19), the measurement noise covariance $R_{k}$ cannot be guaranteed to be a positive definite matrix. In order to ensure that the matrix $R_{k}$ is positive-definite, the adaptive method based on residuals proposed by [47] is used in this paper as follows:

(21) $${\tilde{C}}_{k}=E\left(e_{k}e_{k}^{T}\right)=R_{k}-H_{k}P_{k}^{-}H_{k}^{T}$$

(22) $$R_{k}=E\left(e_{k}e_{k}^{T}\right)+H_{k}P_{k|k-1}H_{k}^{T}$$

Based on the memory index $\alpha_{k}$, adjust the process noise and observation noise covariance matrices $Q_{k}$ and $R_{k}$ by weighted update, so that they reflect the current state change of the system. Introduce the memory index adaptive estimation fairly $R_{k}$ is as follows:

(23) $$R_{k}=\alpha_{k}R_{k-1}+\left(1-\alpha_{k}\right)\left(e_{k}e_{k}^{T}+H_{k}P_{k|k-1}H_{k}^{T}\right)$$

The method of literature [1] is used to reason $Q_{k-1}$. Based on (11) and (12), the state noise variance $Q_{k-1}$ is obtained by using innovation as follows:

(24) $$Q_{k-1}=E\left(W_{k}W_{k}^{T}\right)=K_{k}{\tilde{y}}_{k}{\tilde{y}}_{k}^{T}{}_{}K_{k}^{T}$$

After introducing the memory index $\alpha_{k}$, the adaptive estimate $Q_{k}$ is obtained as:

(25) $$Q_{k}=\alpha_{k}Q_{k-1}+\left(1-\alpha_{k}\right)\left(K_{k}{\tilde{y}}_{k}{\tilde{y}}_{k}^{T}{}_{}K_{k}^{T}\right)$$

By algorithmically obtaining the noise covariance at the current time, we can use it to update the system state and its error covariance at the next time. The whole procedure of the proposed adaptive methodology is shown in Algorithm 1. **Algorithm 1** Adaptive Kalman Filtering based on Memory index1.Inputs: *A*, *H*, $Q_{k}$, $R_{k}$, ${\hat{X}}_{0}$, $P_{0}$, $\alpha_{0}$2.**for** *k* = 1 to *N* **do**3. Prediction step:4. ${\overset{⌢}{X}}_{k|k-1}=A{\hat{X}}_{k|k-1}$5. $P_{k+1|k}=AP_{k|k}A^{T}+Q_{k}$6. Calculate information entropy:7. $S_{k}$ = 08. **while** *i* < *n* **do**9.   *i* = *i* + 110.   By observing data or state vector calculated value of $\mu_{i}$and $\sigma_{i}^{2}$11.   $p_{i}=\frac{1}{\sqrt{2\pi }\sigma_{i}}\exp\left(-\frac{{\left(\beta_{i}-\mu_{i}\right)}^{2}}{2\sigma_{i}^{2}}\right)$12.   $S_{i}=-p_{i}\log p_{i}$13.   $S_{k}=S_{k}+S_{i}$14. **end while**15. **return** $S_{k}$16. Adjustment step:17. $\alpha_{k}=\frac{1}{1+\eta_{k}S_{k}}$18. ${\tilde{y}}_{k}=Z_{k}-H_{k}{\hat{X}}_{k|k-1}$19. $e_{k}=Z_{k}-H_{k}{\hat{X}}_{k|k}$20. $R_{k}=\alpha_{k}R_{k-1}+\left(1-\alpha_{k}\right)\left(e_{k}e_{k}^{T}+H_{k}P_{k|k-1}H_{k}^{T}\right)$21. $Q_{k}=\alpha_{k}Q_{k-1}+\left(1-\alpha_{k}\right)\left(K_{k}{\tilde{y}}_{k}{\tilde{y}}_{k}^{T}{}_{}K_{k}^{T}\right)$22. **end for**

## 4. Collision Warning Strategy

### 4.1. Fusion of Sensors in Space and Time

The millimeter-wave radar and the camera have different spatial locations and sampling frequencies, which lead to inconsistent information about the same target. To achieve effective fusion of the millimeter-wave radar data and the camera image data for forward target recognition, the two sensors need to be calibrated in space and time.

#### 4.1.1. Spatial Fusion of Radar and Camera

Spatial fusion is the process of aligning the data from the millimeter-wave radar and the camera, which are two sensors with different coordinate systems and units. It involves two steps: coordinate transformation and projection. Coordinate transformation converts the target coordinates from the radar system to the world system, which is a common frame for both sensors. Projection maps the target coordinates from the world system to the image system, which is the pixel-based frame of the camera. The details of each step are as follows:

Step 1: The radar polar coordinate is converted to the radar Cartesian coordinate. These formulas are as follows:

(26) $$\{\begin{array}{c} x_{r}=r\cos\phi \cos\theta \\ y_{r}=r\cos\phi \sin\theta \\ z_{r}=r\sin\phi \end{array}$$

where *r* is the distance from the point to the radar origin, *θ* is the azimuth angle of the point in the horizontal plane, and *ϕ* is the elevation angle of the point in the vertical plane.

Step 2: The millimeter-wave radar coordinate system is converted to the camera coordinate system. This can be achieved by using the outer parameter matrix, which is a transformation matrix describing the relative position and attitude relationship between the two coordinate systems. The extrinsic parameter matrix can be estimated by a calibration method. The transformation form is as follows:

(27) $$\left[\begin{array}{c} x_{w}\\ y_{w}\\ z_{w}\\ 1 \end{array}\right]=\left[\begin{array}{cc} R & T\\ 0 & 1 \end{array}\right]\left[\begin{array}{c} x_{r}\\ y_{r}\\ z_{r}\\ 1 \end{array}\right]$$

where ***R*** is a 3 × 3 rotation matrix representing the rotation transformation of the radar coordinate system with respect to the world coordinate system, and ***T*** is a 3 × 1 translation vector representing the translation transformation of the radar coordinate system with respect to the world coordinate system.

**Figure 9:**
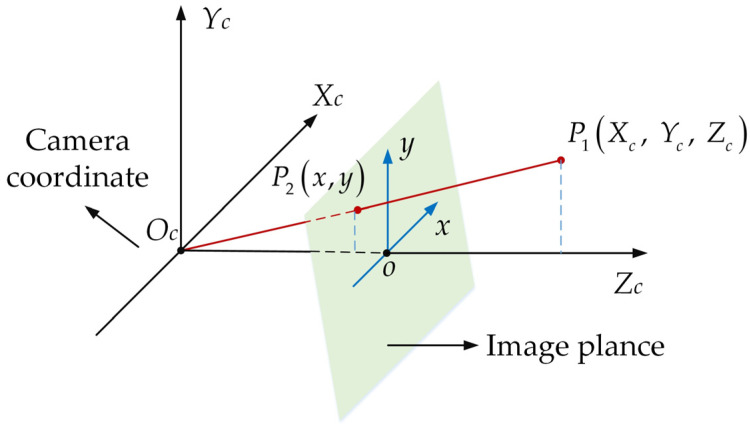
The process of transformation by using the similar triangle principle.

Step 3: The camera coordinate system is converted to the pixel coordinate system. According to the linear model of the camera, any point in the camera coordinate system can be transformed into its corresponding point in the image coordinate system by using the similar triangle principle, as shown in Figure 9. Suppose there is a point $P_{1}\left(X_{c},\;Y_{c},\;Z_{c}\right)$ in the camera coordinate system, and this point is projected to $P_{2}\left(x,y\right)$ in the image coordinate system, then the following transformation formula is obtained:

(28) $$Z_{C}\left[\begin{array}{c} x\\ y\\ 1 \end{array}\right]=\left[\begin{array}{cccc} f & 0 & 0 & 0\\ 0 & f & 0 & 0\\ 0 & 0 & 1 & 0 \end{array}\right]\left[\begin{array}{c} X_{C}\\ Y_{C}\\ Z_{C}\\ 1 \end{array}\right]$$

where *f* is the focal length of the camera, $X_{c},\;Y_{c},\;Z_{c}$ are the coordinates of the points in the camera coordinate system in the world coordinate system.

**Figure 10:**
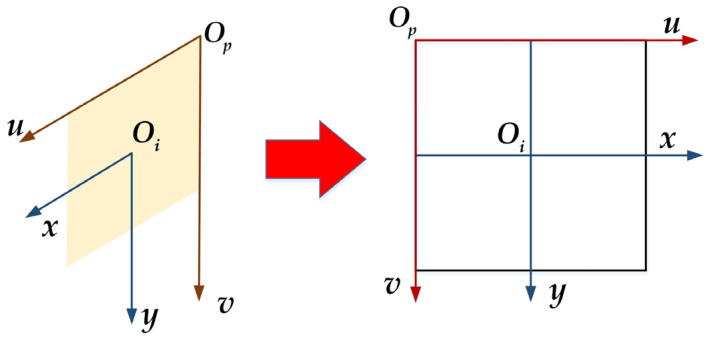
Transformation between the image pixel coordinate system and the image physical coordinate system.

Step 4: The physical coordinate system of the image is converted to the pixel coordinate system. As shown in Figure 10, suppose that a point on the imaging plane has its coordinates in the image coordinate system for $O_{i}=\left(x,\;y\right)$, in pixel coordinates, the coordinates of $O_{p}=\left(u,\;v\right)$. The conversion between them can be conducted using the following formula:

(29) $$\left[\begin{array}{c} u\\ v\\ 1 \end{array}\right]=\left[\begin{array}{ccc} \frac{1}{dx} & 0 & u_{0}\\ 0 & \frac{1}{dy} & v_{0}\\ 0 & 0 & 1 \end{array}\right]\left[\begin{array}{c} x\\ y\\ 1 \end{array}\right]$$

where $\left(u_{0},\;v_{0}\right)$ is the center point in the image pixel coordinate system, that is, the pixel point corresponding to the origin of the image physical coordinate system, and *dx* and *dy* represent the physical dimensions of each pixel along the *u* and *v* axes.

#### 4.1.2. Time Fusion of Radar and Camera

**Figure 11:**
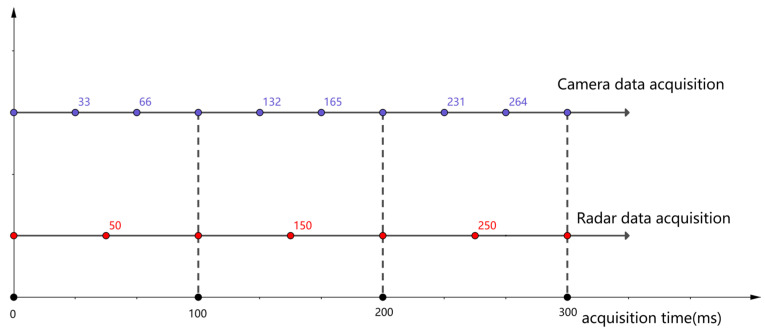
Radar and camera data time synchronization.

Time fusion refers to the synchronization of data from millimeter-wave radar and cameras in time to ensure data consistency and accuracy. To achieve temporal fusion of the millimeter-wave radar and the vision sensor, we synchronize their sampling frequencies according to the slower one. The millimeter-wave radar has a target update rate of 20 Hz, while the vision sensor has a capture rate of 30 Hz. Therefore, the time interval between each frame of the radar data is 50 ms, and that of the vision sensor is 33.3 ms. As shown in Figure 11, we make the radar and the vision sensor sample data simultaneously every 100 ms, which is the least common multiple of their time intervals. For example, when the radar obtains a frame of data at 100 ms, the vision sensor also completes an image capture at the same time, ensuring temporal synchronization for multi-sensor fusion.

### 4.2. Decision Level Fusion with Intersection over Union Ratio

#### 4.2.1. Formation of Regions of Interest

**Figure 12:**
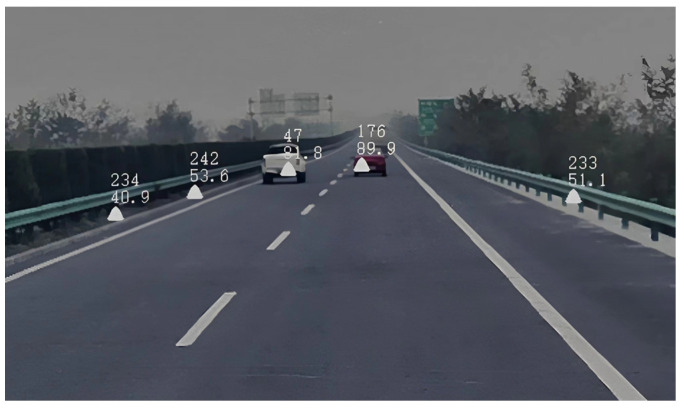
The radar is used to detect target projections in the image.

The data output of the millimeter-wave radar detection is mapped onto the image by spatial transformation, as shown in Figure 12. A rectangular box with a width of 2.6 m and a height of 2.0 m is constructed as the Region of Interest (ROI) around the target point measured by the millimeter-wave radar, according to the vehicle shape size specified by the national standard [48].

#### 4.2.2. Information Fusion Based on IoU

The radar output results and the visual output results are fused by using the Intersection over Union (IoU) method [49], which is a measure of the overlap between the predicted and the ground truth bounding boxes. The IoU method determines whether the detected target exists by comparing the ratio of the intersection area and the union area of the two bounding boxes. The area of ROI formed by the target detected by radar is $S_{R}$, and the target identified by the improved YOLOv5 visual detection algorithm will also generate an ROI area; its area is $S_{C}$. Based on the visual ROI region and the radar ROI region, the IOU is as follows:

(30) $$\mathrm{IOU}=\frac{S_{C}\cap S_{R}}{S_{C}\cup S_{R}}$$

where $S_{C}\cap S_{R}$ is the area of the intersection part of the radar ROI and visual ROI, and $S_{C}\cup S_{R}$ is the area of their union part. The threshold of the IOU is set to 0.4, or 0.6.

IoU is a commonly used metric to measure the similarity between two bounding boxes. IoU-based fusion methods aim to combine information from different sensors or modalities based on IoU values. However, it is not easy to choose the IoU threshold for information fusion, as it depends heavily on the specific object detection task and dataset. There is no one-size-fits-all recommended threshold for IoU because different thresholds may have different impacts on the precision and recall of fusion results, as well as on false positives and false negatives. The common threshold used in practice is 0.5, which means that the IoU between the predicted frame and the ground truth frame must be at least 0.5 to be considered a true positive detection. However, the application scenario of this study is a small, long-range target in a highway driving environment. This threshold may not be suitable for the application scenario of this study, as it may be too strict and lead to many false negatives. Therefore, according to empirical observations and road experiments, this paper chose to use two IoU thresholds of 0.4 and 0.6 for information fusion. It was found that these two thresholds can strike a good balance between precision and recall and also reduce false positives and false negatives due to occlusions, truncations, and misalignments.

**Figure 13:**
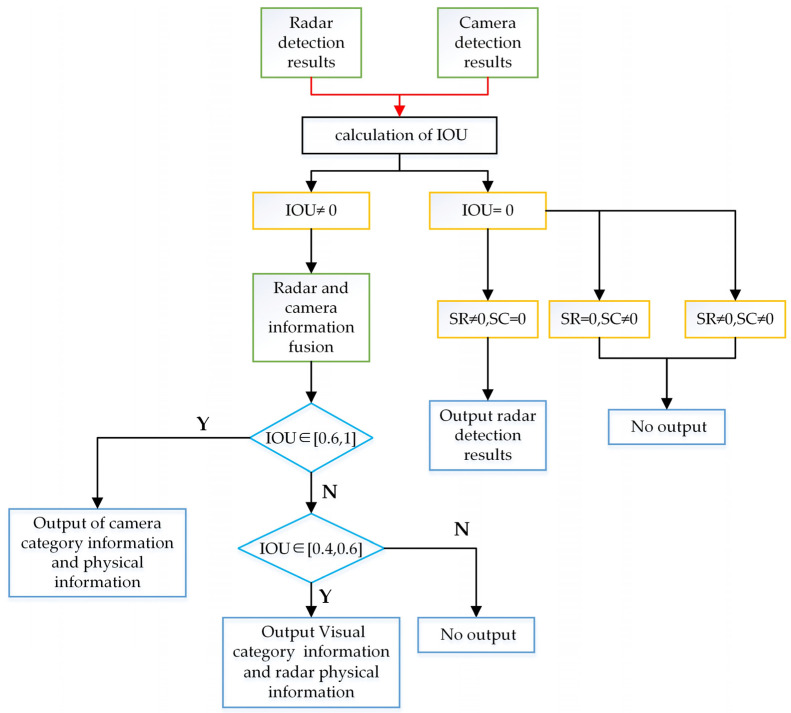
IOU discrimination process.

If the IOU is in the range [0.6, 1], it indicates that both sensors can detect the same target with high accuracy. Because the data projected on the image by the radar is biased, the output results are the category and physical information of the target detected by the camera. If the IOU is in the range [0.4, 0.6], it indicates that there is some error between the results of the two sensors detecting the target. In this case, the physical information of the target detected by the radar is more accurate than that of the camera. Therefore, the results of the output target are, respectively, the visual detection category and the physical information detected by the radar. If IOU = 0, there are three cases: no millimeter wave radar ROI, no visual ROI, and both millimeter wave radar ROI and visual ROI exist without any intersection between them. For Case 1: IOU = 0, $S_{C}=0,S_{R}\neq 0$, the camera is affected by the complex environment, and the target physical information results detected by the radar are output. For case 2: IOU = 0, $S_{C}\neq 0,S_{R}=0$, the visual detection is wrong, and the detection result is not output. For case 2: IOU = 0, S = 0, $S_{C}\neq 0,S_{R}\neq 0$ there is a large deviation between the visual detection results and the radar detection results, and the detection result is not output. The IOU discrimination process is shown in Figure 13.

### 4.3. Forward Collision Warning Strategy

In this section, we propose a forward collision warning (FCW) strategy based on the minimum safety distance (MSD), which is designed to provide timely and suitable warning to the driver under various driving scenarios and conditions. The main idea of this strategy is to compute the MSD between the host vehicle and the preceding vehicle using their kinematic and dynamic parameters and contrast it with the distance output by the fusion algorithm. If this distance is lower than the MSD, a warning is triggered to alert the driver of a potential collision. The MSD calculation model is based on the following assumptions:

- The host vehicle and the preceding vehicle are moving in the same direction on a straight road segment.
- The host vehicle and the preceding vehicle have similar braking capabilities and deceleration rates.
- The driver of the host vehicle has a constant reaction time and follows a constant headway policy.
- The driver of the preceding vehicle applies a constant deceleration when braking.

**Figure 14:**
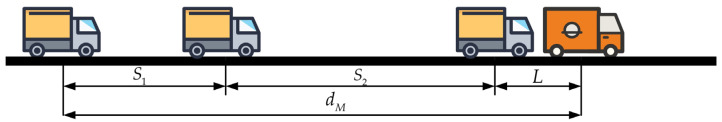
MSD model of the vehicle in front at rest.

Based on these assumptions, we calculate the MSD between two vehicles in three cases:

Case 1: When the front vehicle is stationary. The driver of the rear vehicle recognizes the danger when the vehicle in front abruptly stops. After the reaction time *t*, the driver applies the brakes. Then, the rear car decelerates until it stops. To avoid colliding with the leading vehicle, the distance between the leading vehicle and the rear vehicle must be at least $d_{M}$. As shown in Figure 14, the MSD in this case is modeled as follows:

(31) $$d_{M}=S_{1}+S_{2}+L=v_{1}t+v_{1}{}^{2}/2a_{1}+L$$

where $S_{1}$ is the distance traveled by the rear vehicle during the driver’s reaction time, $S_{2}$ is the distance traveled by the rear vehicle during the period from deceleration to stopping, $v_{1}$ is the initial velocity of the following vehicle, $a_{1}$ is the maximum deceleration of the following vehicle, *t* is the driver’s braking reaction time, and *L* is the length of the vehicle.

**Figure 15:**
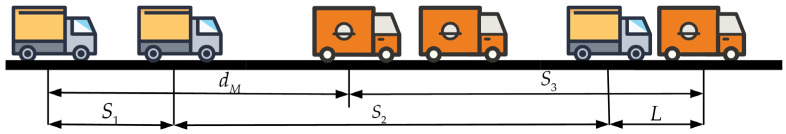
MSD model of the front vehicle at constant speed.

Case 2: When the front vehicle is moving at a constant speed. The MSD for this case is illustrated in Figure 15. In contrast to case 1, in this case, the rear vehicle, after a reaction time *t*, begins to decelerate until it matches the speed of the front vehicle and then keeps its speed to follow the front vehicle. The MSD model for this case is as follows:

(32) $$d_{M}=S_{1}+S_{3}-S_{4}+L$$

where $S_{3}$ is the distance traveled by the rear vehicle in the process of changing from deceleration to uniform speed, $S_{4}$ is the distance traveled by the front vehicle during the uniform speed process. These two distances are as follows:

(33) $$S_{3}=\frac{v_{1}^{2}-v_{2}^{2}}{2a_{2}}$$

(34) $$S_{4}=v_{2}t+v_{2}\left(\frac{v_{1}-v_{2}}{a_{2}}\right)$$

where $v_{2}$ is the speed of the front vehicle, $a_{2}$ is the acceleration of the rear vehicle as it decelerates. Combining (32)–(34), the safety distance model of the front vehicle at constant speed can be obtained as follows:

(35) $$d_{M}=\frac{v_{1}^{2}-v_{2}^{2}}{2a}-\frac{v_{2}^{2}-v_{1}v_{2}}{a}+(v_{1}-v_{2})t+L$$

**Figure 16:**
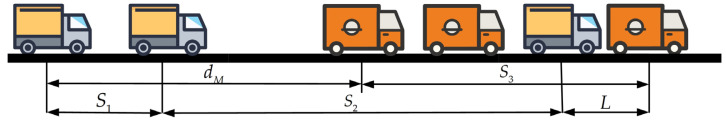
MSD model of the front vehicle driving at a reduced speed.

Case 3: When the vehicle in front slows down. This case is shown in Figure 16. The driver realizes that the vehicle ahead suddenly slows down. In order to avoid collisions with the leading vehicle, the MSD between the leading vehicle and the following vehicle is as follows:

(36) $$d_{M}=v_{1}t+\frac{v_{1}^{2}-v_{3}^{2}}{2a_{3}}-\frac{v_{2}^{2}-v_{3}^{2}}{2a_{4}}+L$$

where $v_{1}$ is the speed of the rear vehicle, $v_{2}$ is the speed of the front vehicle, $v_{3}$ is the speed of the two vehicles at exactly collision, $a_{3}$ is the deceleration of the following vehicle, and $a_{4}$ is the deceleration of the leading vehicle.

The MSD for the above three different working conditions is difficult to meet in the actual situation. Therefore, this paper corrects and optimizes the MSD according to the driver’s personality, emotion, attention, fatigue, and other factors to make it more in line with the actual situation. The model can better adapt to different driving scenarios and improve the safety and efficiency of intelligent driving.

Firstly, the driver’s behavioral characteristics greatly affect the driver’s reaction time. Therefore, according to the driver’s behavioral characteristics, the correction coefficient can be set for dynamic adjustment of reaction time. The formula for the value of the correction coefficient is as follows:

(37) $$k_{t}=f(P,E,A,F)=1-0.01\times (P+E+A+F)$$

where $k_{t}$ is the correction factor for reaction time, *P* is the driver’s personality score, *E* is the driver’s emotional score, *A* is the driver’s attention score, and *F* is the driver’s fatigue score. These ratings generally range from 0 to 10, with higher ratings indicating less favorable driving conditions.

On the other hand, road conditions also affect the safety distance model. According to the geometric characteristics, physical characteristics, and traffic characteristics of the road, the speed, acceleration, and braking acceleration of the vehicle in the minimum safety distance model are modified. According to the curvature, slope, degree of wetness, traffic flow, and other factors of the road, some correction coefficients are set as follows:

(38) $$\begin{array}{l} k_{c}=1-0.01\times R\\ k_{s}=1-0.02\times S\\ k_{f}=0.5+0.5\times \mu \\ k_{d}=1-0.01\times D \end{array}$$

where $k_{c}$ is the curvature correction coefficient, $k_{s}$ is the slope correction coefficient, $k_{f}$ is the friction coefficient correction coefficient, $k_{d}$ is the vehicle density correction coefficient, *R* is the road curvature radius, *S* is the road slope angle, *μ* is the road friction coefficient, and *D* is the road vehicle density.

In this paper, these correction coefficients are substituted into the safety distance model of three different working conditions, and the optimized safety distance model a can be obtained as follows:

(39) $$d_{M}=\left\{ \begin{array}{l} k_{c}v_{1}k_{t}t+\frac{{\left(k_{c}v_{1}\right)}^{2}}{2ak_{s}k_{f}}+k_{d}L\\ k_{c}{}^{2}\left(\frac{v_{1}^{2}-v_{2}^{2}}{2ak_{s}k_{f}}-\frac{v_{2}^{2}-v_{1}v_{2}}{ak_{s}k_{f}}\right)+(v_{1}-v_{2})k_{c}t+k_{d}L\\ k_{c}v_{1}k_{t}t+k_{c}{}^{2}\left(\frac{v_{1}^{2}-v_{3}^{2}}{2a_{3}k_{s}k_{f}}-\frac{v_{2}^{2}-v_{3}^{2}}{2a_{4}k_{s}k_{f}}\right)+k_{d}L \end{array}\right.$$

## 5. Experimental

In this section, the SKBAM visual inspection algorithm as well as the improved adaptive Kalman filter algorithm are experimented with, and their performance is verified. Finally, the decision-level fusion algorithm for vision and radar was tested on the experimental vehicle and compared with the conventional fusion algorithm.

### 5.1. Improved YOLOv5 Experiment

#### 5.1.1. Vehicle Datasets for Visual Detection

To evaluate the improved YOLOv5 algorithm, this paper used the CompCars dataset [50], the UA-DETRAC dataset [51], and other car-related datasets and selected some of them to form the dataset of the ablation experiment. The vehicle targets in the dataset were categorized into four types, namely big cars, tiny cars, trucks, and special cars. There are a total of 4026 images in the dataset, including 2426 images in the training set and 1600 images in the test set, and the size of the images was resized to 640 × 640.

#### 5.1.2. Experimental Environment and Parameter Configuration

The experiments were conducted on a computer running the Windows 11 operating system. The computer was equipped with an Intel i7-13700 processor and an Nvidia GeForce RTX 1650 graphics card. The programming language used is Python 3.7.0, and the deep learning framework is PyTorch 1.12.1. GPU acceleration was enabled by installing CUDA 11.7. The model was trained for 100 epochs with a learning rate of 0.001 and a batch size of 32.

To evaluate the performance of YOLOv5 with the SKCAM attention module, the accuracy and speed of the model need to be measured. In this paper, it is measured by indicators such as mean precision (*mAP*) recall and precision, which is the average square of precision values at different recall levels. Speed can be measured by metrics like frames per second (FPS), which refers to the number of images that a model can process in one second.

*Precision* is a measure of how accurate the predictions are compared to the actual outcomes. The formula for *Precision* is:

(40) $$Precision=\frac{TP}{TP+FP}$$

where *TP* (true positive is the correct detection of the object) is defined as the number of samples whose model prediction is positive and whose true label is also positive. *FP* (false positive) is defined as the number of examples that the model predicts to be positive, but the true label is negative.

*Recall* is the ratio of true positives to the sum of true positives and false negatives, where a false negative is a missed prediction. It is expressed as follows:

(41) $$Recall=\frac{TP}{TP+FN}$$

where *FN* (false negative) is defined as the number of examples that the model predicts to be negative, but the true label is positive.

Mean Average *Recall* (*mAR*) can be computed at different IoU thresholds, such as 0.5, 0.75, or 0.5:0.95. It is expressed as follows:

(42) $$AP=\int_{0}^{1}P(R)dR$$

(43) $$mAP=\frac{1}{N}\sum_{i=1}^{N}AP_{i}$$

#### 5.1.3. Results and Discussion

**Figure 17:**
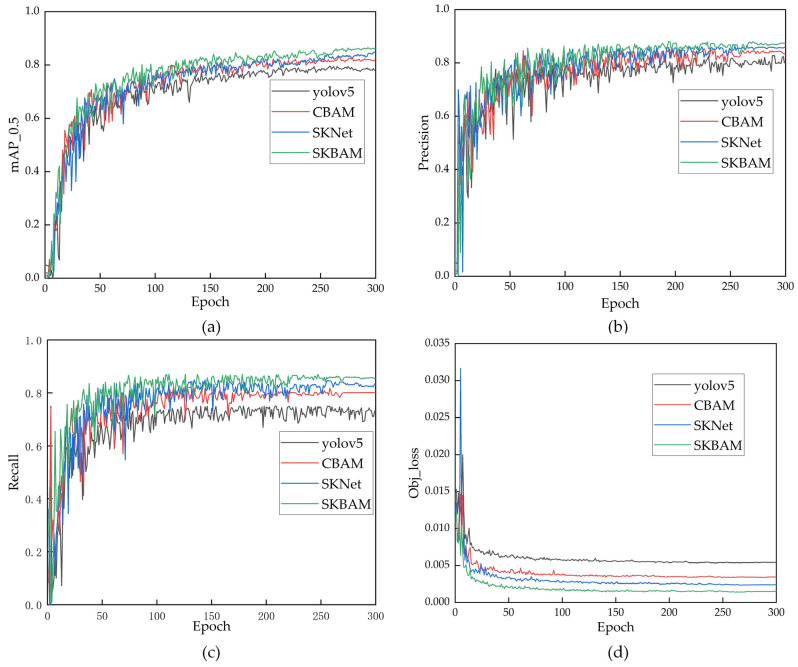
Training results of YOLOv5 and models adding various attention mechanism modules to the vehicle dataset (**a**) is the change curve of mAP_0.5, (**b**) is the change curve of precision, (**c**) is the change curve of recall, and (**d**) is the change curve of Obj_loss.

In this section, we conducted an ablation experiment to evaluate the effectiveness of the SKBAM module proposed in this paper. We compared the original YOLOv5 model with three variants: YOLOv5 + CBAM, YOLOv5 + SKNet, and YOLOv5 + SKBAM. We added all the attention modules to the same position in the network. Figure 17 shows the experimental results.

Figure 17a–c shows the precision, recall, and *mAP* of the four models during 300 training epochs. The curves demonstrate that the original YOLOv5 and the three variants with attention modules achieve fast improvement and gradual stabilization in their performance. Among them, YOLOv5 + SKBAM outperforms other models in all metrics. Figure 17d shows the comparative loss functions of different models. It is evident that YOLOv5 + SKBAM has the lowest loss value and converges quicker than other models.

The trained models were also evaluated on a benchmark dataset, and the results are shown in Table 1. It is evident that adding attention modules to YOLOv5 improves the detection accuracy to some degree. In particular, compared to YOLOv5 + CBAM, YOLOv5 + SKBAM boosts the precision by 4.09%, the *mAP* by 4.08%, and the recall by 5.25%. It also enhances the detection speed from 63 frames per second to 67 frames per second. Likewise, compared to YOLOv5 + SKNet, YOLOv5 + SKBAM improves the precision by 1.46%, the *mAP* by 1.48%, and the recall by 1.97%. However, it slightly lowers the detection speed from 73 frames per second to 67 frames per second. This is because SKBAM considers both spatial and channel attention, which increases the detection accuracy but also the computational cost. Based on these experimental results, we conclude that YOLOv5 + SKBAM outperforms other models and demonstrates the effectiveness of the proposed module.

### 5.2. Improved AEKF Experiment

**Figure 18:**
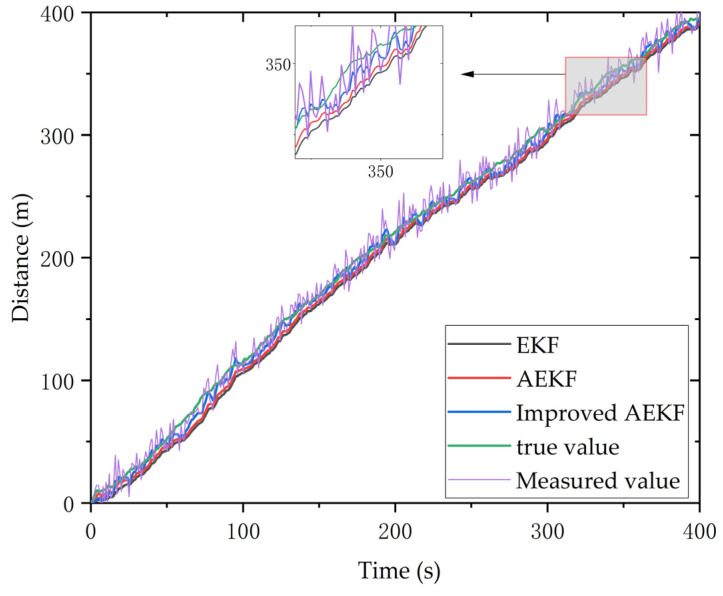
Response results of different algorithms when tracking a target with temporal variations.

In this section, we evaluate the reliability of the proposed AEKF algorithm. We conducted experiments in the test center to simulate the specified motion state and path of the test vehicle. Then the radar tracked vehicle state data using the traditional EKF algorithm, and the proposed AEKF algorithm was recorded. Figure 18 shows the response results of the EKF algorithm (black line), the AEKF algorithm proposed in [52] (red line), and our improved AEKF algorithm (blue line) for tracking targets with time changes. The real value in the figure is the integration of the actual vehicle speed and time recorded by the vehicle speed sensor, which is obtained by mathematical calculation. The measured value is the one measured by the millimeter-wave radar sensor. From the local image of Figure 18, it can be observed that the proposed algorithm is closer to the true value than the other two algorithms.

**Figure 19:**
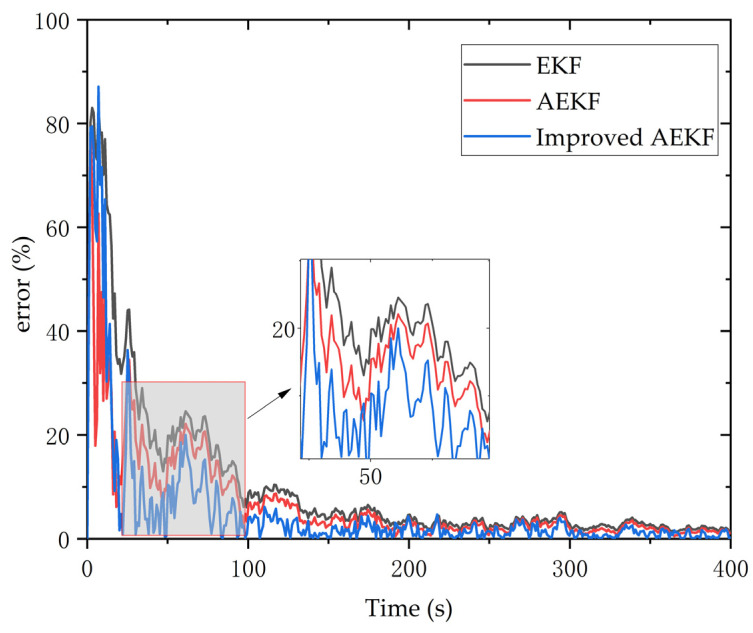
Comparison of errors between estimated and actual values of different algorithms.

In this paragraph, we illustrate the advantages of the proposed algorithm more intuitively by calculating the error between the estimated value and the actual value of the algorithm at each instant. The results are shown in Figure 19, which compares the EKF error (black line), the AEKF error (red line), and the improved AEKF error (blue line). It is evident from the figure that the estimated value of the algorithm converges to the actual value, and the error decreases until it is nearly zero. The local enlarged figure shows that the improved AEKF algorithm proposed in this paper has smaller error and better convergence than the other two algorithms in most time intervals. Based on the error data between the estimated value and the actual value, we use the root mean square error (RMSE) to further compare the performance of each filtering algorithm, which is evaluated as follows:

(44) $$\mathrm{RMSE}=\sqrt{\frac{1}{n}\sum_{i=1}^{n}{\left({\hat{y}}_{i}-y_{i}\right)}^{2}}$$

where ${\hat{y}}_{i}$ represents the optimal estimate processed by the algorithm and $y_{i}$ represents the true value. The calculation results show that the RMSE value of the traditional algorithm EKF is 10.147830, the RMSE value of AEKF is 7.73227, and the RMSE value of the improved AEKF algorithm is 4.98201. The comparison shows that the error between the optimal estimation value and the actual value of the algorithm used in this paper is smaller, and the accuracy is higher.

### 5.3. Collision Warning Experiment

**Figure 20:**
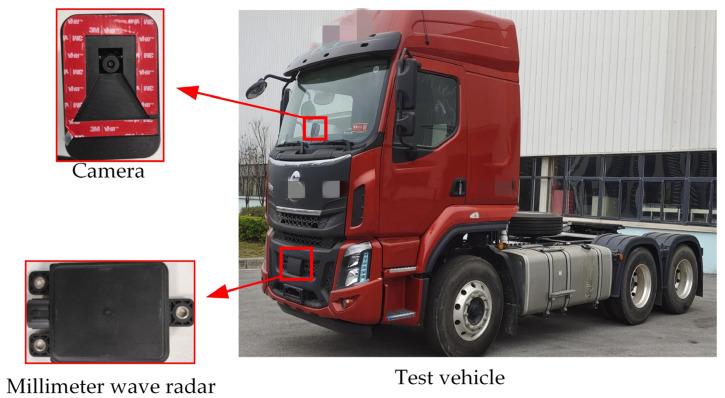
Location of the millimeter-wave radar and camera installation.

To evaluate the accuracy and effectiveness of the proposed fusion warning method, we conducted road tests with a test vehicle under various weather conditions (sunny day, sunny night, cloudy day, cloudy night, rainy day, and rainy night) and road environments (different road surfaces and different working scenarios). The millimeter wave radar used in the experiment was ANNGIC FR55F, which operates in the frequency range of 76 GHz~77 GHz. The image recognition sensor was the ANNGIC FV-12M, which produces images with a resolution of 1280 × 1080 pixels and a frame rate of 30 frames per second. Figure 20 shows the installation location of the test vehicle and the sensors.

**Figure 21:**
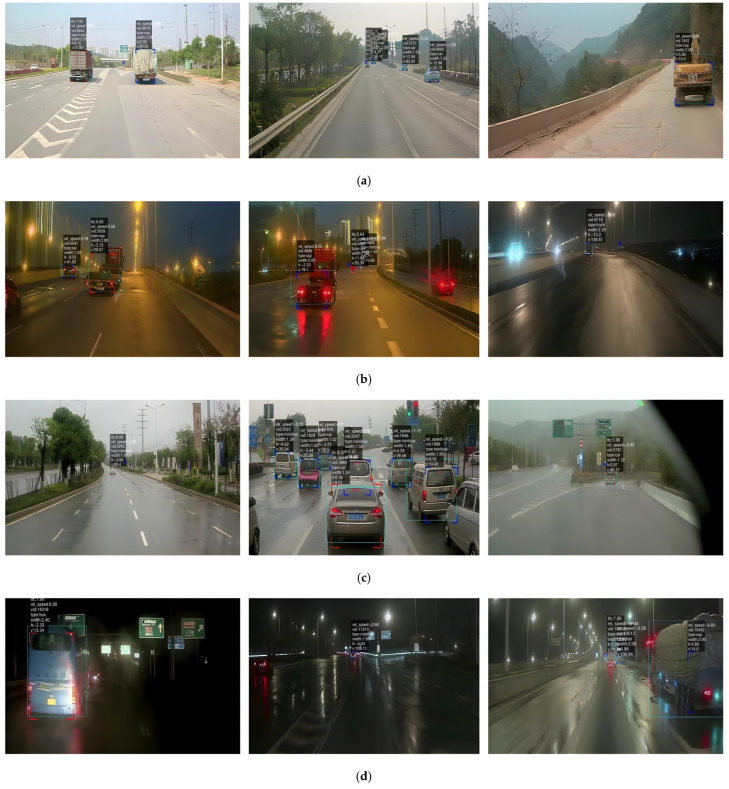
Effect of early warning experiments based on the fusion of millimeter-wave radar and cameras in different environments (**a**) is the detection result of the road environment in the daytime, (**b**) is the detection result of the road environment at night, (**c**) is the result of testing in a rainy road environment, and (**d**) is the result of testing in the rainy at night road environment.

This paper presented experiments in different road environments, including various road surfaces, working conditions, and weather situations. The experiments in this paper were conducted on the road where the vehicles are traveling normally. The mileage of the test vehicle was about 6000 km. Due to the large mileage of the experiment, the experimental procedure included various obstacle vehicles and other obstacles in different states. The result of the fusion of millimeter-wave radar and cameras is illustrated in Figure 21. Figure 21a–d shows the detection of vehicle targets in the road environments of day, night, rainy day, and night rain, respectively. The green box in each figure indicates the visual detection results, while the red box and blue box indicate the detection results of millimeter-wave radar. The physical information of the detected target was recorded above the bounding box, which helps to assess whether the test vehicle is at risk of colliding with the target. The time to collision (TTC) was calculated based on the preset safe distance and speed. In these experiments, the red detection box represents a collision warning for the front vehicle, and the blue detection box represents a safe vehicle that does not require a warning.

In Figure 21, our fusion warning algorithm correctly identifies vehicle targets in different working conditions, different models, and different environments and accurately detects dangerous vehicles. In order to prove the influence of the two algorithms on the experimental results, this paper analyzed the data using one-way analysis of variance (ANOVA). Firstly, the independent variables were set as two kinds of traditional algorithms and this paper’s algorithm, and the dependent variable was the experimental data. Two hypotheses were designed: the original hypothesis and the alternative hypothesis. The original hypothesis was defined as different algorithms having no effect on the experimental results, and the alternative hypothesis was defined as different algorithms having an effect on the experimental results. The significance level (α) was set at 0.05. In this paper, an ANOVA was performed using R. The ANOVA test revealed that the statistic F was 6.355 with a *p*-value of 0.0408. The *p*-value was less than the level of significance. Therefore, this meant that the original hypothesis could be rejected, and it was concluded that the algorithm had a significant effect on the experimental results. To demonstrate the effectiveness of the collision warning algorithm in this paper, the proposed algorithm is compared with the traditional algorithm. The traditional fusion algorithm is defined as follows: The EKF filtering algorithm is used to track the target for radar, and the YOLOv5 target recognition algorithm is used for vision. The conditions remain the same, except that the two algorithms are different. The two algorithms were tested based on the same scene environment and mileage, and the comparative experimental results are shown in Table 2. Table 2 compares the experimental results of the two methods under four different weather conditions, using accuracy rate, missed alarm rate, and false alarm rate as the evaluation metrics. The proposed algorithm has a slightly lower accuracy rate (1.3615%) than the traditional algorithm in the rainy night environment, but it outperforms the traditional algorithm in other environments, especially in the sunny night environment. Overall, the proposed algorithm achieves an accuracy rate of 93.193%. The false alarm rate and missed alarm rate of the proposed algorithm, compared to the traditional algorithm, are reduced by 11.619% and 15.672%, respectively. The traditional method uses the YOLOv5 algorithm for vision detection, which has a low recognition rate at night and causes a high false alarm rate. At the same time, the traditional method also uses the EKF algorithm for radar tracking, which is easily affected by environmental noise and leads to a high missed alarm rate. In contrast, the proposed method uses a vision algorithm that emphasizes more on the target feature information, which lowers the missed detection rate to some degree, and an improved AEKF tracking algorithm that adaptively updates the environmental noise, which enhances the accuracy of radar target tracking.

## 6. Conclusions

In this paper, a forward collision warning strategy based on millimeter-wave radar and vision fusion is proposed to solve the problem of high false alarm and omission rates of existing multi-sensor fusion algorithms in complex weather and road environments. The strategy improves the visual detection target algorithm and the millimeter-wave radar tracking target algorithm, respectively, and effectively fuses the improved visual detection results with the radar tracking results.

On the one hand, the two sub-modules of the CBAM attention mechanism are improved, and the SKBAM attention mechanism is designed. Then, it is added to the YOLOv5s model to improve the accuracy of detecting vehicle targets. The experimental results show that the detection accuracy of the proposed model is higher than other algorithms with attention mechanisms, especially 3.11% better than the original YOLOv5 algorithm.

On the other hand, in order to improve the target tracking accuracy of the Kalman filter algorithm. In this paper, an information entropy-based memory index adaptive Kalman filter algorithm is proposed, which can adaptively adjust the noise covariance according to the change of the system state and optimize the performance of EKF. Simulation proves that the RSME index of the algorithm is 5.16582 lower than the original EKF.

Finally, based on the IoU and the minimum safe distance model, a decision-level fusion warning strategy that fuses visual and radar detection results is proposed. Forward collision warning experiments were conducted under different weather and road conditions. The experimental results show that the proposed algorithm improves the accuracy of collision warning and reduces false alarm and omission rates compared with the traditional algorithm. The detection accuracy reaches 97.323% in clear weather and 93.193% in mixed weather. Compared with the existing fusion warning strategy, the false alarm rate is reduced by 11.619% and the missed alarm rate is reduced by 15.672%. In future work, the strategy will be improved to increase accuracy in nighttime rain scenarios.

## Figures and Tables

**Table 1:** Comparison of detection results between YOLOv5 and adding various attention mechanism modules.

Name	*Precision* (%)	*mAP* (%)	*Recall* (%)	FPS
YOLOv5	80.01	78.27	73.17	82
YOLOv5 + CBAM	83.27	82.11	80.09	63
YOLOv5 + SKNet	85.90	84.71	83.37	73
YOLOv5 + SKBAM	87.36	86.19	85.34	67

**Table 2:** Comparison of experimental results between the traditional fusion early warning method and the method proposed in this paper.

Day or Night Environment	Sunny or Rainy	Algorithm	Alarms (Times)	Missed Alarms (Times)	False Alarms (Times)	Accuracy (%)	Missed Alarm Rate (%)	False Alarm Rate (%)
Day	Sunny	Tradition	937	13	23	96.211	1.387	2.455
		Ours	926	8	17	97.323	0.864	1.836
	rainy	Tradition	2376	83	156	90.281	3.493	6.566
		Ours	2039	67	121	91.073	3.286	5.934
Night	Sunny	Tradition	763	39	10	93.890	5.111	1.311
		Ours	973	33	13	95.427	3.392	1.336
	rainy	Tradition	386	31	8	90.647	8.031	2.073
		Ours	393	27	18	89.286	6.870	4.580
aggregate		Tradition	4462	166	197	92.156	3.720	4.415
		Ours	4331	135	169	93.193	3.117	3.902

## Data Availability

Due to the nature of this research, participants of this study did not agree for their data to be shared publicly, so supporting data is not available.
